# Review of Recent Inkjet-Printed Capacitive Tactile Sensors

**DOI:** 10.3390/s17112593

**Published:** 2017-11-10

**Authors:** Ahmed Salim, Sungjoon Lim

**Affiliations:** School of Electrical and Electronics Engineering, College of Engineering, Chung-Ang University, 221, Heukseok-Dong, Dongjak-Gu, Seoul 156-756, Korea; ahmedsalim789@gmail.com

**Keywords:** inkjet printing, tactile sensors, capacitive sensing, paper, flexible polymer, glass

## Abstract

Inkjet printing is an advanced printing technology that has been used to develop conducting layers, interconnects and other features on a variety of substrates. It is an additive manufacturing process that offers cost-effective, lightweight designs and simplifies the fabrication process with little effort. There is hardly sufficient research on tactile sensors and inkjet printing. Advancements in materials science and inkjet printing greatly facilitate the realization of sophisticated tactile sensors. Starting from the concept of capacitive sensing, a brief comparison of printing techniques, the essential requirements of inkjet-printing and the attractive features of state-of-the art inkjet-printed tactile sensors developed on diverse substrates (paper, polymer, glass and textile) are presented in this comprehensive review. Recent trends in inkjet-printed wearable/flexible and foldable tactile sensors are evaluated, paving the way for future research.

## 1. Introduction

It is predicted that by 2018 consumers will be using 1.8 billion units of smart phones and 447 million tablets, which shows the great demand for touch screens [1]. The applications of tactile sensing include the capacitive Windows button on the Microsoft Surface Pro, touch screens (mobiles, television, tablets and notepads) and interactive boards. Advanced applications of tactile sensing can be seen in robotics (touch detection and artificial skin) and gaming (object detection through touch panels and touch tables). Previous trends and advancements in tactile sensors, ranging from academic designs to commercial products, are presented in Table 1. Since 2000, there has been a rapid increase in research and development related to tactile sensors. 

Tactile sensing is accomplished through human touch and interpreted by the brain. The sense of touch makes movements accurate and stable, the loss of which leads to meaningless activity. Human skin consists of cutaneous receptors that, with the help of the kinesthetic modality, can sense texture, softness, vibration, temperature, roughness, shape, size, composition and force [2]. Intelligent interaction, intentional manipulation and desired responsiveness were the earliest driving forces for the development of tactile sensors. A touch event is described as an interaction between a hand, a finger, or an object with a tactile sensor [3]. Proximity sensing (noncontact sensing) requires a more sensitive sensing mechanism than touch sensing; nonetheless, the underlying principle is similar to that of touch sensing. Vision-based sensing, which is another type of noncontact sensing, is not considered in this article. In 1980, Harmon described the specifications of tactile sensors and set guidelines for subsequent designers [4]. The most important properties of all practical sensors are:

(1) Stable and hysteresis-free behavior
(2) Monotonic response (better if linear)
(3) Conformable interface, robust and inexpensive

The spatial resolution, sensitivity and dynamic range are three additional characteristics.

Tactile sensors can be classified according to their transduction method: resistive, capacitive, piezoelectric, or optical [5]. A resistive sensor consists of a conductive material whose resistance changes when pressure is applied. This change corresponds to the deformation, which alters the density of the material under applied pressure [5]. The simple design of sensor, low cost, good sensitivity, low noise and compact design of readout circuitry are their advantages [6]. The inherent drawbacks are high power consumption, the short life-span of the materials and hysteresis [6]. Piezoelectric materials (e.g., quartz) are non-conductive materials which generate charges proportionally to the applied-force to the sensor [6]. Their fast and linear response make them useful for dynamic force sensing [6]. Capacitive sensing is the most widely used mechanism after resistive sensing [7]. A basic capacitive sensor consists of two conducting plates separated by an insulating layer. The separating distance and/or the effective area of the plates is changed by shifting their relative position using an applied force [8]. Commercially available capacitive touch sensors such as RoboTouch, DigiTacts (Pressure Profile Systems 2007), DiamondTouch (Circle Twelve Inc., Framingham, MA, USA, 2008), Touche´ (Disney Research 2012) and iPodtouch (Apple Inc., Cupertino, CA, USA., 2008) are famous products/patents that use the capacitive touch-sensing mechanism [8]. Optical sensors utilize optical reflection originating from media having different refractive indices [8]. However, being bulky and expensive, they are not particularly attractive. 

## 2. Inkjet-Printing Technology

### 2.1. Inkjet Printing 

There are numerous printing techniques in use, such as screen printing, spray printing, micro-transfer printing, gravure printing, laser printing, manual coating and lithography. Screen printing is a mature printing technology wherein a screen is utilized as a stencil and ink layers are applied on the printing surface. It produces a high-quality pattern; however, the low resolution, required design complexity, inefficient ink consumption and insufficient control of the ink thickness are limiting factors in contemporary usage, particularly for radiofrequency (RF) applications [9]. Laser-printing technology offers a higher resolution (<10 μm) than inkjet printing and roll-to-roll printing; however, it cannot be used for cost-effective mass production [10]. Photolithography is a high-resolution technique that have been compatible with rigid substrates; and using some modifications and/or optimization it has also been successfully applied to print the patterns on flexible and easily-damageable substrates [11,12,13]. However, it is a multi-step (time consuming) and inefficient process that requires mask preparation as a prerequisite. In addition, it demands a particular environment (clean room), requires hazardous chemical solutions for etching and consumes more material [14]. To overcome these issues, alternative technologies are investigated. 

Inkjet printing, which commenced in the 1970s, is a rapidly emerging technique for direct patterning via material deposition based on a layout designed in software. In the inkjet-printing process, a print head (carrying an ink-filled cartridge) horizontally moves back and forth and tiny droplets of ink are propelled through a micrometer-sized nozzle head [11,12,13]. The materials to be deposited are usually in the form of colloidal dispersions or chemical solutions. There are three major actuation mechanisms of the inkjet nozzle head: thermal, piezoelectric and electro hydrodynamic. Inkjet printing is a versatile printing technology that not only reduces the manufacturing cost but also reduces the manufacturing steps. Additional attractive features of inkjet printing include its compatibility with elastomers, high efficiency, maskless and additive printing process [14]. Inkjet-printing technologies are compared, and it is emphasized that for research purposes and to check the validity of innovative ideas, low cost inkjet-printing is an appropriate choice [15]. In addition to obvious advantages for military applications, low cost inkjet-printing is a suitable choice for “small-cheap-disposable” sensors [15].

### 2.2. Substrate Materials

Inkjet-printing technology facilitates the utilization of a variety of unconventional substrates for various designs [14,15,16,17,18,19,20,21,22]. In this subsection, we discuss the attractive features of unconventional substrates.

The printing of electronics on paper commenced in the 1960 [20] and the first inkjet machine was invented in the 1970s [9] but major developments could not occur until the late 1990s. Photo paper/inkjet paper is glossy, coated on one or both sides and has fast-drying properties. It is free of additives (whitening/bleaching agents), which reduces the possibility of interference [21]. The paper substrate has many advantageous features; e.g., it is inexpensive, eco-friendly, lightweight, expandable, foldable in three dimensions and available everywhere [22].

Elastomer substrates tend to provide flexibility and bendability, which are essential requirements for wearable devices, artificial skin and flexible touch screens. Polydimethylsiloxane (PDMS) is widely used as a flexible substrate [23]. For a detailed comparison of polymer substrates, [24] can be seen. 

Textiles/fabrics remain a primary choice for clothing-based wearable electronics because of their compliance with human skin [25] and other benefits (lightweight, long-lasting and able to twist) [26,27]. They are not yet considered a stable material for inkjet printing [28]. The benefits and limitations of materials commonly used as the sensor-substrate are compared in Table 2 [21].

To facilitate simulation and modelling of inkjet-printed designs, it is important to accurately plugin the design parameters. The geometry and dielectric properties of substrates greatly impact on the system’s performance. The permittivity and loss tangent of commonly used substrates are compiled in the Table 3.

### 2.3. Conductive Inks and Sintering

For an ink to produce stable droplets and reliable conductive patterns, it should withstand certain physical and chemical conditions, the details of which are presented in [26]. Organic and inorganic materials can be utilized as printable materials; however, the former are preferred because of their flexibility, low cost and simple synthesis, although the latter exhibit higher electrical performance [26]. To achieve a low-cost and simplified printing process, four types of ink formulation (nanoparticle inks, conducting organic inks, catalyst inks and reaction inkjet system) have been used to realize metal tracks [33]. The first two of these are the most widely used inks. We briefly discuss these with an emphasis on the constituents of these inks and their suitability for specific substrates/applications.

Nanoparticle ink comprises particles of metal or semiconductor oxides mixed with a desired solvent (mostly organic compounds [22]). These inks have been printed onto all kinds of substrates. The metal loadings range between 20% and 70% and typically reach 30% [34]. The inks are dried and converted into a solid after ejection through the nozzle. Copper (Cu), silver (Ag), gold (Au) and nickel (Ni) nanoparticles are commonly utilized conductive inks to develop patterns through inkjet-printing [35]. Conductive Ag nanoparticles are the most widely used inkjet-printing ink. It has the highest conductivity among all metals and is significantly less expensive price than Au, which are the main reasons why Ag is the first choice as the ink material for printed electronics [34]. Although Cu-based inks are far less expensive than Ag, their lower stability necessitates stringent post processing (in a vacuum or protective environment) for avoiding oxidation.

Materials such as Ag nanoparticles, single-walled C nanotubes (SWCNTs) dissolved in water/ethanol, SWCNT/RuO_2_ nanowires and CNT/PEDOT:PSS have been inkjet-printed on various textiles to realize flexible wearable electronics [10,28]. Thin sheets of conductive (e.g., Ag Nanowire) and composite materials have also been in use [36].

Sintering is the most common post-processing technology used after the deposition of ink through inkjet printing. The conductivity of nanoparticles is significantly lower than that of the bulk material. During sintering, the metallic nanoparticles diffuse into each other (reducing the porosity), creating a continuous connectivity of trace and increasing the electrical conductivity [20,32,33]. Although thermal sintering requires a high temperature and a long processing time [34], it is an easily adaptable post-processing technique. It only requires an oven/heating plate with a temperature between 100–300 °C for a duration of more than 30 min [37].

Conductive polymers were introduced to facilitate the formation of electrical patterns on flexible polymer substrates, as these substrates cannot be treated using conventional thermal sintering, which is imperative for metallic-nanoparticle ink-based patterns [3]. In addition, conductive polymers have good mechanical stability and adhesion to plastic substrates. PEDOT:PSS and polyaniline are among the most studied polymers for ink formulation [38]. Generally, printed patterns from conductive polymer-based inks exhibit less conductivity than metallic nanoparticle-based inks [33]. Stretchable Ag conducting ink (Dupont PE872) is considered an elegant solution for stretchable and wearable electronics. Screen-printed microwave designs using this ink have been reported [36,39]. This ink sustains its stretchability and can endure up to 100 wash cycles [40]. 

Indium tin oxide (ITO) exhibits a high transmittance (90%) and low sheet resistance (10 Ω/square) and has been considered the industry standard for optoelectronics applications, such as liquid crystal displays, solar cells, OLEDs and touch sensors integrated into display screens [41]. The scarcity of indium, high cost, inherently fragile nature and the fact that ITO is mostly limited to lithographic printing are considerable drawbacks [42] suggesting ITO cannot remain as the first-choice deposition material for optoelectronics applications in the future. ITO inkjet-printed sensors are also fabricated [43], where a solution of ITO nanoparticles dispersed in ethanol is inkjet-printed (UJ200, Unijet, UniJet Co. Ltd, Songnam, Korea) to realize transparent electrodes on a glass substrate. On the other hand, PEDOT:PSS-based conductive and transparent inks are attracting attention from researchers yet are unpopular in inkjet printing because of their insolubility in common solvents [14]. Nevertheless, a composite of PEDOT:PSS (showing high solubility) is considered to be one of the alternatives to ITO [42].

To characterize an inkjet-printed pattern, the conductivity and permittivity of nanoparticle inks and dielectric inks, respectively have been given in Table 4. To model a design close to the real one, these values are important to know and they have a profound impact on the system’s performance.

### 2.4. Resolution of Inkjet Printing

In order to get a proper drop placement which directly leads to high quality inkjet-printed design, the aerodynamics, shape and size of droplets must be controlled. The lower drop spacing yields higher conductivity of printed tracks, for instance 60 µm, 30 µm and 15 µm drop spacing would generate the better conductive tracks, respectively [46]. The resolution of inkjet printing is an important parameter that mainly depends on the ink volume propelled by the inkjet-printing machine [9]. The width of the printed tracks is limited by the resolution of the printer [47], for example the resolution of Dimatix is 10–30 µm depending on drop volume [48]. The constituents of the ink, the substrate, the sintering process and the voltage waveform applied to the jetting nozzles affect the resolution and accuracy of the inkjet-printing process [9]. The best resolution achieved using inkjet-printing technology is searched out after consulting several research studies, as shown in Table 5. A resolution as high as 2 µm has been achieved in inkjet printing [49,50].

### 2.5. Challenges and Strategies of Inkjet Printing

There are certain challenges associated with the inkjet-printing process. For a successful material deposition, the inks should have a high electrical conductivity, resistance to oxidation and good adhesion to the substrate. The conductivity of these inks can be controlled according to the concentration of metallic nanoparticles and the bonding structure after solidification [22]. To increase the electrical conductivity, using a highly viscous ink appears to be option. If a highly viscous ink is deposited via droplets, either the homogeneity is compromised or clogging/blockage occurs at the nozzle opening [32]. To ensure the smooth and uniform dispersion of nanoparticles, the agglomeration of the conducting ink must be prevented [24] and inks with the proper viscosity and a solvent with a low evaporation rate are recommended [52]. Clogging can be resolved by manual cleaning and/or dipping the nozzle in a cleaning solution or distilled water **[53,54]**. Nevertheless, researchers have invented a sophisticated technique to avoid nozzle clogging: “catalyst ink,” which is combined with metal particles for electroless deposition and reduces the dependence on the sintering process [22]. When conventional metallic nanoparticles have been used in electroless plating (auto-catalytic plating), the notion of “catalyst ink” is used. In electroless plating only one electrode exists without external power source and it undergo metal reduction with the help of a reducing agent, a complexing agent and/or additive(s). Metal particles aggregate on the catalytic active surface and this process of metal reduction and growth is continued [55]. 

Because of imperfections such as, pores, cracks and surfactants etc., the conductivity of inkjet-printed material suffers, and it is much less than the bulk conductivity of the metal [56]. To ensure high conductivity close to that of bulk metal, electroless deposition has been introduced, wherein a catalyst ink is used [22,56]. 

Commonly used metals such as Ag, Cu and Au tarnish even Ag is the metal that most readily oxidizes even if expose to dry and ambient conditions, due to the presence of detrimental compounds in the air like carbonyl sulphide and hydrogen sulphide. In case of electrochemical sensor, the conductivity is degraded because of oxidation and it must be considered. In order to avoid or reduce the tarnish, some passivation treatments have been applied in the past, for instance deposition of polymers or nickel on the Ag electrodes [35]. Depending on application and required sensitivity, Au is a good choice after Ag [35].

Despite providing a novel platform for inexpensive, portable and simple designs, paper as a substrate poses considerable drawbacks, i.e., it absorbs moisture and is non-fire-retardant; therefore, it is unsuitable for inkjet printing. Low-viscos inks not only provide inadequate electrical conductivity (discussed above) but are also inclined to be absorbed by a paper substrate. In order to add hydrophobicity and sustain high-temperature post processing without burning, various textures, compositions and thin textile layers(s) are coated onto the paper [9]. In this way permeation, overspreading and in-depth penetration of ink are avoided [22,26,57]. Surface treatment also makes the paper fire-retardant. Another issue with paper substrate arises after the sintering process. There is no doubt the sintering process makes sure high conductivity, however, it reduces the lifetime of paper substrate [22]. Fortunately, researchers have invented sintering free conductive ink [58]. The gold nanorods coated with certain polymers in a specific composition brought this sintering free ink. They testified its conductivity up to the conductivity of conventional metallic nanoparticle ink and it could retain it (at ambient temperature) even after a year [58].

The decrease in sheet resistance of printing tracks was also observed. Immediately after printing, measured values on Mitsubishi paper and Kodak paper were 0.21 Ω/sq and 1.3 Ω/sq, respectively. After 10 h later the values were 0.19 Ω/sq and 0.28 Ω/sq, respectively [59].

Textiles substrates are porous, compressible, non-robust and moisture-absorbing. Their rough surface deteriorates under inkjet printing and almost no textiles can tolerate long-term thermal curing above 150 °C [26]. On one hand, researchers have synthesized new materials and inks that exhibit little dependence on the post-curing temperature and even sintering-free attributes. On the other hand, alternative technologies for sintering have also been developed and can be useful for flexible substrates that are sensitive to thermal heating [34].

Adhesive materials can also raise issues, which are addressed here. Additives such as polymers, glucose and starch are commonly used in the formation of sticky things (for instance, in glue and adhesive tape) [60]. They can possibly react with ink solutions or undergo undesired changes in their chemical properties during sintering. The composition of these sticky materials can cause false-positive readings for some sensors. Additionally, materials from different brands having the same functionality cannot guarantee reproducibility [21]. It is advised that office materials (adhesive tape, laminated sheets and inkjet-printing materials) must be used after a careful investigation and sophisticated bonding tapes for adhesiveness should be employed as interference layers [21].

## 3. Capacitive Tactile Sensors

**Figure 1:**
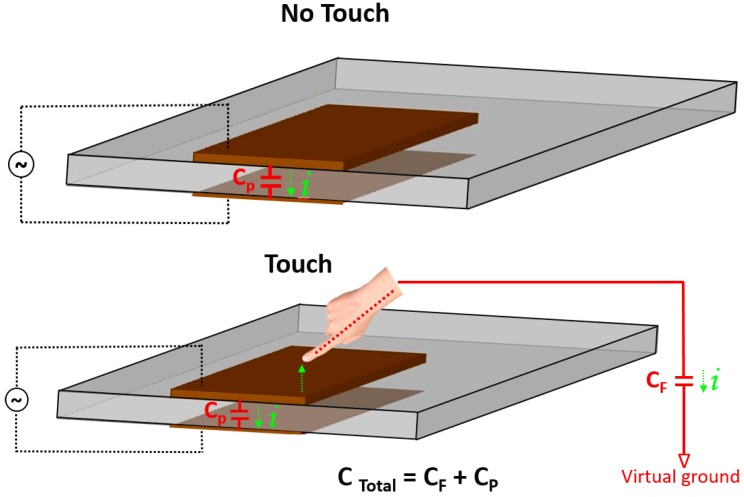
Principle of capacitive sensing.

The principle of a capacitive tactile sensor can be explained according to a two-plate capacitor model. When an object comes in proximity or in direct contact with the capacitor plate, the capacitance of the plate (C_P_) and the capacitance of the human finger/body (C_F_) are parallel and the effective capacitance (C_Total_) increases, as shown in Figure 1. This slight change in capacitance (∆C ~ 1 pF) indicates a touch event [7] and by comparing these capacitance changes with the system threshold, we can determine the coordinates of the object-in-touch [7]. Two or more electrodes on a single substrate or multiple substrates, as well as resonators, have been widely used to realize capacitive tactile sensors.

It is stated that diagonally separated touches may cause false detection, which arises from the intersection of two real points on each axis [1]. Mutual capacitance is believed to solve this issue [1]. In the following section, state-of-the art inkjet-printed tactile sensors developed on various substrates are discussed. The design, sensing phenomena and attractive features are highlighted.

### 3.1. Inkjet-Printed Tactile Sensors on Flexible Substrates

The Internet of Things (IoT) involves several new concepts of network connectivity between physical objects and end users, motivating investigations into new tactile sensors and sensing mechanisms [61]. Flexible pressure sensors and tactile sensors are considered the core of wearable electronics, particularly health-monitoring devices [62]. Therefore, there is great demand for flexible and bendable materials. In this subsection, we discuss inkjet-printed tactile sensors fabricated on flexible substrates (polymer, plastic, paper and textile).

#### 3.1.1. Inkjet-Printed Tactile Sensors on Polymer Substrates

Inkjet/aerosol-jet printed capacitive touch sensors were developed on two-dimensional and three-dimensional polymer substrates [63]. Ag nano-ink (EMD5714, SunChemical, Carlstadt, NJ, USA) was used to print an interdigital capacitor on a thermoplastic substrate (polybutylene terephthalate). The researchers evaluated the touch sensor with a finger (2 s per touch) and detected the change in the capacitance of the two electrodes.

**Figure 2:**
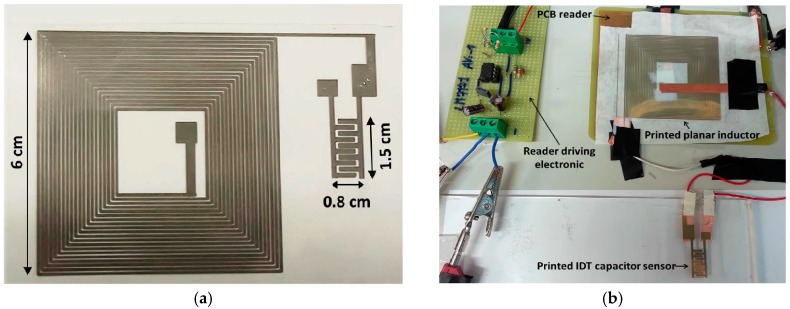
Inkjet-printed LC resonator on a PET substrate, serving as a touch sensor (redrawn from [38]): (**a**) layout; (**b**) complete design of the touch-sensor system (printed LC sensing block coupled to the printed circuit board (PCB)-based contactless, passive reader).

A passive LC resonator on a flexible substrate (polyethylene terephthalate (PET)) was proposed as a touch sensor [38]. Ag nanoparticle ink (Metalon JS-015, NovaCentrix, Austin, TX, USA) was inkjet-printed to realize a planar LC resonator. An inter-digitated capacitive pattern (L × W = 1.5 × 0.8 cm) served as a sensing element with conductive tracks 200 nm thick, as shown in Figure 2. The large size of the reader circuitry (because it was designed for low frequencies of 76 and 146 KHz, as the resonance frequencies correspond to 90 and 60 mm) was its drawback. Nevertheless, its contactless feature and passive readout circuitry make it an attractive choice for battery-less environments.

**Figure 3:**
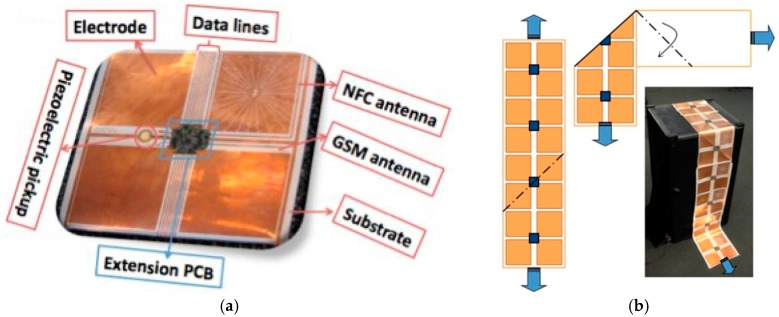
(**a**) A single sensing tile consists of the substrate along with a PCB module (redrawn from [64]). Four different electrodes in each sensing tile transmit four unique signals during footstep detection; (**b**) Demonstration of the folding mechanism for adapting the surface geometry using a single substrate without any cutting or joining.

The sensing floor, which is an interesting application of tactile sensing, was introduced [64]. A sheet of tiles is formed by inkjet-printing a Cu pattern on a flexible plastic substrate, as shown in Figure 3. Four electrodes (120 × 120 mm) for capacitive sensing and two RF antennas (one for cellular GSM detection and another for near-field communication sensing) were printed. The footstep patterns were detected by electrodes embedded in the proposed sensing floor (passive capacitive sensing mode).

**Figure 4:**
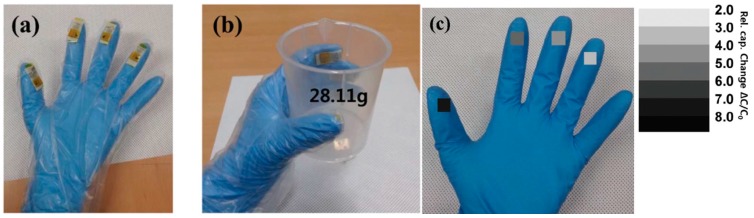
Fingertip grip pressure-sensing device (redrawn from [65]). (**a**) Fingertip grip pressure sensors attached to four fingertips; (**b**,**c**) Holding a plastic beaker with four fingertips; the resultant relative capacitance changes are indicated.

A flexible capacitive pressure sensor with an excellent but fixed sensitivity is proposed [65]. Ag ink (Sigma–Aldrich, St. Louis, MO, USA) was inkjet-printed (DMP 2831, Dimatix Fujifilm, Santa Clara, CA, USA) on a flexible substrate (Arylite, 200 μm thick, Ferrania Corp., Liguria, Italy). A PMMA solution [mixture of PMMA (Sigma–Aldrich) dissolved in propylene glycol methyl ether acetate (PGMEA)] or a poly(vinylpyrrolidone) (PVP) solution [mixture of PVP (Sigma–Aldrich) and poly (melamine-co-formaldehyde) dissolved in PGMEA] was spin-coated on the Ag-printed Arylite substrate to form a dielectric layer. To fabricate pressure sensors, a multiscale-structured electrode was laminated on the top of the dielectric layer and a thin PET film (Toray Corp., Chuo-ku, Japan) was attached on top of the multiscale-structured PDMS to reduce the adhesiveness of the PDMS during the pressure-sensing tests. To perform measurements and characterization, a Cu wire was connected to both electrodes using Ag epoxy. Fingertip grip sensing was characterized, as shown in Figure 4.

**Figure 5:**
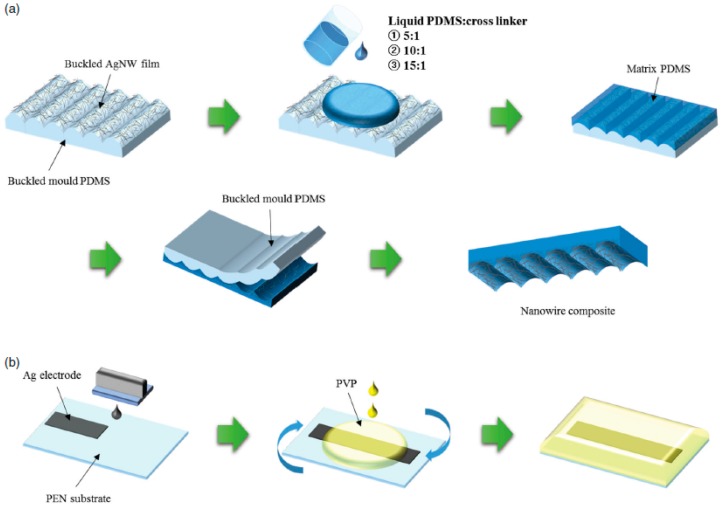

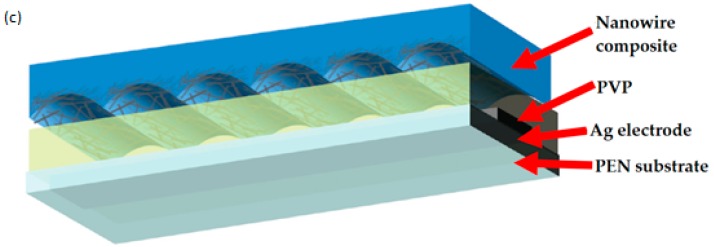
Fabrication process for the capacitive flexible pressure sensor with a nanowire composite and a bottom plane (redrawn from [62]). (**a**) Fabrication process for nanowire composites with different mixing ratios of the PDMS matrix; (**b**) Fabrication process for the bottom plane: the inkjet printing of the Ag electrode onto the PEN substrate and the spin coating of the PVP dielectric layer; (**c**) Layout of the final product.

One year later, the same research group proposed another flexible pressure sensor with a tunable sensitivity, which introduced new avenues for versatile applications (similar to the behavior of human skin) [62]. Ag nanowires (AgNW ink; SLV-NW-90, Blue Nano Inc., Cornelius, NC, USA) were spray-coated on a buckled mold PDMS. Three different formation ratios of the PDMS crosslinker were poured on the AgNW film to form a nanocomposite. The bottom plane was formed using an inkjet-printed Ag electrode on a poly(ethylene2, 6-naphtharate) (PEN) substrate. To perform a bending test, AgNW-embedded PDMS was affixed to the bottom side using Kapton tape and force was applied to the sensor using a bending-test machine. An IMADA force test bed was used to precisely apply vertical pressure using metal and/or plastic weights. A successful characterization of the microstructured AgNW-embedded PDMS confirmed the wavy structure of the nanowire composites, which was independent of the mixing ratio of the PDMS matrix. However, the shape dependence of the buckled structure on the PDMS mixing ratio was confirmed and the nanowire composite with a 10:1 mixing ratio exhibited the greatest change in its relative capacitance value. The fabrication steps and final layout of the capacitive flexible pressure sensor are shown in Figure 5. 

**Figure 6:**
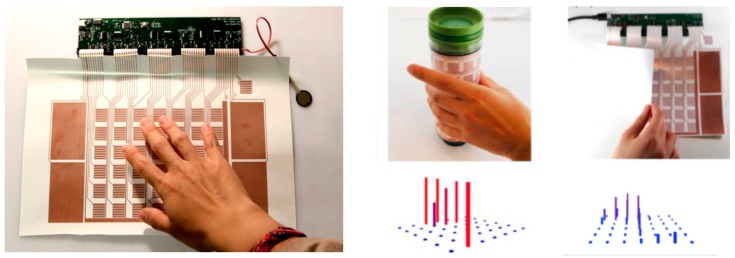
PrintSense: a new development in multi-modal sensing arrays, printed as a single layer on a flexible substrate and connected to a customized hardware module (**left**). Capacitive touch sensing, proximity sensing, galvanic skin response (i.e., resistive sensing) and curved sensing are supported (redrawn from [66]).

**Figure 7:**
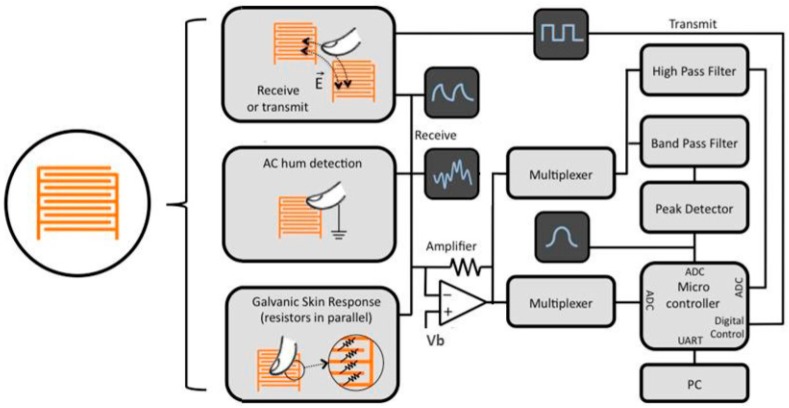
PrintSense: Three different signal-detection schemes (redrawn from [66]). From top to bottom: active transmit-and-receive capacitive sensing for proximity and folding detection, alternating-current hum detection for touch and proximity detection and galvanic skin response.

“PrintSense” is an interesting sensing device capable of multimodal flexible interaction [66]. It is effective in various sensing scenarios, such as touch and hover-based as well as curved and deformable interfaces. It is a hybrid structure consisting of an inkjet-printed array of interdigitated electrodes (single layer) on a flexible substrate and customized circuitry to process electrode signals generated by finger touches, as shown in Figure 6. To increase the detection range of proximity capacitive sensing, two additional sensing modes were included, as shown in Figure 7. 

A capacitive force sensor, i.e., a parallel-plate capacitor with PDMS (Sylgard 184, Dow Corning, Wiesbaden, Germany) as the dielectric, was proposed for artificial skin [67]. One electrode was realized using Kapton (Pyralux, DuPont, Wilmington, DE, USA), with the top surface covered by a Cu laminated sheet. The other electrode was inkjet-printed using a solution of Ag nanoparticle ink (DGP 40LT-15C, ANP Co., Pleasanton, CA, USA) dissolved in triethylene glycol monoethyl ether. They characterized different thicknesses of the PDMS dielectric as well as the printed layers and analyzed the PDMS swelling phenomenon (coffee-ring effect). The change in the capacitance divided by the change in the force was defined as the sensitivity and its highest value was 3 pF/N.

Piezoresistive artificial skin capable of tactile sensing was proposed [3]. PEDOT: PSS ink (CleviosTM, Heraeus Precious Metals GmbH, Hanau, Germany) was inkjet-printed (JETLAB 4-XL, Microfab Technologies, Inc., Plano, TX, USA) on a Cu-PDMS composite substrate. The conductivity of inkjet-printed patterns may suffer under elongation/bending. Secondly, the weak adhesion between the polymeric-based functional material and the metalized electrode may lead to peeling/detachment effects. Conventional plasma treatment cannot be employed to overcome these detrimental effects; therefore, the optimized direct patterning of electrodes on an already plasma-treated composite material is realized.

The fabrication of an instant inkjet-printed flexible sticker using commercially available inks and a conventional office printer was proposed [59]. An interdigitated capacitive electrode functions as a sensor and can detect even the exact touch location when a slight change in the effective permittivity near its interface is induced. The accessible, low-cost and rapid printing technique is claimed to be viable for designing custom shape patterns on both flexible substrates (polymer and paper) and conventional (rigid) substrates.

Low-cost polymer substrates having a temperature limitation (approximately 200 °C) are unsuitable for high-performance flexible electronics. To overcome this issue, technical collaboration was initiated between ORNL and NovaCentrix [68]. An Ag/Cu Metalon ink (NovaCentrix)-based inkjet-printed capacitive touchpad is realized on various temperature-sensitive flexible substrates (PET and polyimide). The novelty was the unique photonic curing process (using PulseForge^®^ tools) to heat the thin films with the high-intensity, short-duration (250 μs) light pulses required for thin-film densification, recrystallization and annealing. This process is selective to inkjet-printed patterns and does not damage the main substrate material or other integrated circuits.

#### 3.1.2. Inkjet-Printed Tactile Sensors on Paper Substrates

Photo paper offers remarkable advantages as a sensor-substrate, which were presented in Section 2. Here, we discuss several paper-based tactile sensors fabricated using inkjet-printing technology. 

**Figure 8:**
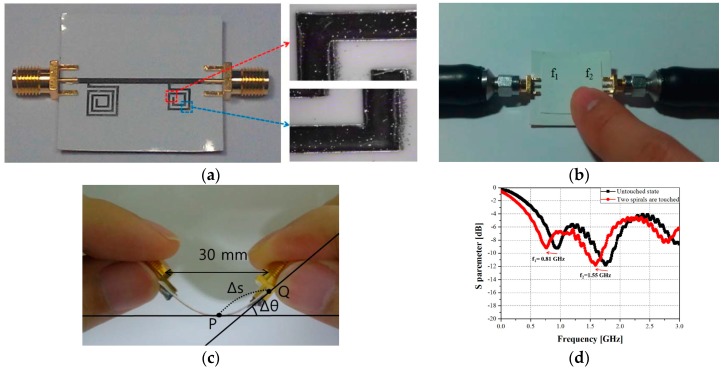
Touchpad: inkjet-printed spiral resonator on photo paper (redrawn from [69]). (**a**) Fabricated prototype of the touchpad (**b**) when one spiral is touched, (**c**) side view of the bent touchpad at a specific curvature ratio and (**d**) measured S21 when both spiral resonators are touched.

In [69], an inkjet-printed touchpad based on spiral resonators is proposed. Its principle phenomenon relies on the detection of changes in the resonance frequency of the spiral resonator due to a finger approaching the touchpad and causing electromagnetic coupling. It can detect one or two fingers even if the fingers are in close proximity of the sensor surface. The touchpad was designed on Kodak photo paper, using ANP Ag Jet 55 LT-25C Ag nanoparticle ink with a Dimatix DMP-2831 printer, as shown in Figure 8. To ensure a high conductivity, three layers of Ag nanoparticles were printed and then sintering (2 h at 120 °C) was conducted. The measured S21 shows that distinctive resonance frequencies were obtained when both spirals touched individually and simultaneously, as shown in Figure 8d.

In [70], a multi-key touch interface based on resistive touch sensing is proposed on photographic paper. Voltage-divider circuitry is in action when a finger is brought in contact and it acts as a close switch, which otherwise remains open. The researchers evaluated the touchpad with 10, 15 and 20 touch contacts, yielding an accuracy of 99%, 93% and 91%, respectively. A touch detection rate of 154 touches per minute was reported. They used an Epson WF30 printer, along with NBSIJ-MU01 ink and paper from Mitsubishi Imaging (Rye, NY, USA).

In [71] a multi-touch capacitive sensor is proposed, which can be customized in shape and size. They used Ag conductive ink and an inkjet printer to print conductive layers and electrodes on paper. They demonstrated their touchpad idea using various topologies and sizes and demonstrated the robustness of the sensing device even after it was cut. 

In [72], an interactive paper serving as a paper-based touch sensor via inkjet printing and conductive ink is proposed. The complete design process—from design and printing to system integration—is described and one can fabricate it without knowing technical details. The researchers also presented a recording option and logic demonstration; however, the design process was lengthy and slow.

A capacitive touchpad consisting of nine touch buttons is proposed, which can detect a change in the effective capacitance when the impedance is altered by a finger or touch pen in contact with the sensor surface [73]. An EPSON printer was used to print Ag nanoparticle ink (Metalon JS-B25P, Novacentrix, Austin, TX, USA) on a photo paper (Kodak, Rochester, NY, USA). They evaluated the sensor performance using a test setup (ARDUINO board, a demultiplex chip and an amplifier) and reported the distinguished capacitance values as 288–236 and 340–564 pF for the untouched and touched states, respectively. The touchpad exhibited a height sensitivity of 1.88 mm^−1^ for h < 0.2 mm and an area sensitivity of 0.02082 mm^−2^ for A < 35 mm^2^.

**Figure 9:**
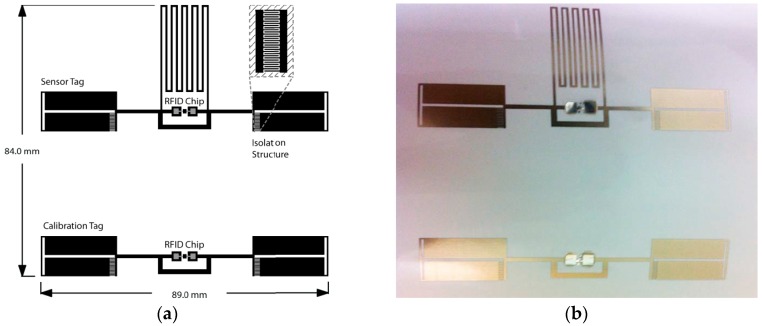
RFID touch sensor (redrawn from [74]): (**a**) layout of chip-based touch sensor and (**b**) fabricated prototype of inkjet-printed RFID touch sensor.

A wireless touch and proximity sensor is proposed [74]. It consists of two inkjet-printed antennas (one for sensing and one for reference), as shown in Figure 9. The matching of the sensing antenna changes when a finger is touched or comes in proximity. The sensing and calibration antenna exhibited a similar return loss and a relative shift of 60 MHz was observed when a touch event is detected.

A capacitive touch control pad was fabricated via a hybrid approach on a paper, kapton and glass substrate [75]. Here, a Dimatix DMP-2831 printer was used to inkjet-print 40 wt. % Ag nanoparticle ink (Silverjet DGP HR, ANP, Bugang-myeon, Sejong, Korea) with a 10-pL cartridge on paper (Powercoat 230, Arjowiggins, Stamford, CT, USA) and polyimide Kapton (ISOAD TAPE 7004, DuPont, Wilmington, DE, USA). Nozzle platen (at 40 °C) was selected to assist the vaporization of the volatile solvent, yielding the best droplets on the surface. Three different materials were tested to connect surface mount devices to the inkjet-printed pads: a Silverjet ink compound, which is also used for the inkjet-printed pattern; Ag epoxy (CircuitWorks Conductive Epoxy CW2400, Chemtronix, Kennesaw, GA, USA); and solder flux (Qualitek, Sn 97% Cu 3%). All three soldering materials were characterized on each substrate and evaluated with regard to the lowest contact resistances: solder for Kapton with 0.12 Ω and AgNP for paper and glass with 0.27 Ω and 0.37 Ω, respectively.

It is not simple to integrate buttons with a touch sensor/screen. Until 2012, there was only one patent for capacitive buttons on posters, which describes a methodology for receiving input/keystrokes on paper-based substrates. However, some research articles (for instance, [69]) discuss the effect of adding one or more extra layer(s) on top of paper-based touch sensors and characterize the touch response. In [76], the researchers characterized two different types of capacitive buttons in relation to their usage on a paper-based keypad (with 10 individual keys).

#### 3.1.3. Inkjet-Printed Tactile Sensors on Textile Substrate

**Figure 10:**
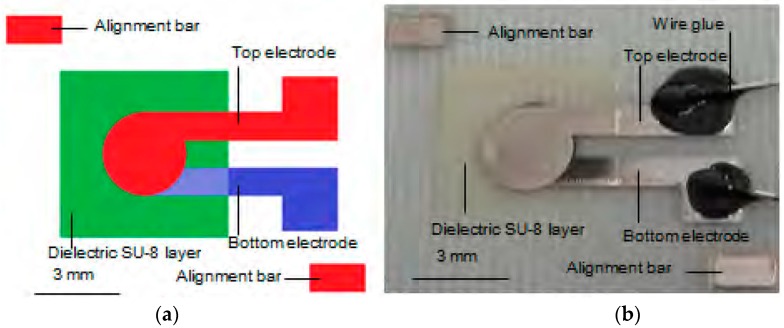
Top view of the fabric-based all-inkjet-printed flexible capacitor: (**a**) schematic view generated using L-Edit software; (**b**) fabricated prototype (redrawn from [25]).

Capacitors are fundamental elements in proximity and touch sensors, in addition to playing various roles in electronic circuits. In [25], an inkjet-printed textile (65/35 polyester cotton)-based flexible parallel-plate capacitor was designed. The 65/35 polyester cotton (Klopman Ltd., Frosinone, Italy) was pretreated with polyurethane (Fabink-UV-IF1, Smart Fabric Inks Ltd., Southampton, UK) using a screen-printing technique. This interface layer was designed to reduce the surface roughness of the original fabric. Ag conductive ink (U5714, SunChemical, Carlstadt, NJ, USA) was inkjet-printed (Dimatix DMP-2831, FUJIFILM Dimatix, Inc., Lebanon, NH, USA) with a cartridge having a 10-pL droplet volume to fabricate top and bottom Ag conductive electrodes and the resulting system was cured at 150 °C for 10 min. The resolution of the printed pattern was controlled using a Dimatix printer, by adjusting the droplet spacing between 5 and 254 μm. To prevent “pattern bleeding,” which occurs because of an excessive volume per unit area, a droplet spacing that is too small must be avoided. The middle layer (dielectric part) was also inkjet-printed using a solution consisting of PVP and a poly(melamine-co-formaldehyde) methylated solution in 1-hexanol. A photoinitiator called bis (4-tert-butylphenyl) iodonium triflate (DtBPIT) was added to this dielectric solution and the resulting mixture was treated in a UV chamber (Mercury bulb, 160 nm λ) for 100 s. In this research work, they referred their previous work; an all inkjet-printed capacitor on textile, in which SU-8 served as dielectric layer [44], as shown in Figure 10.

### 3.2. Inkjet-Printed Tactile Sensors on Rigid Substrates

#### Inkjet-Printed Tactile Sensors on Glass Substrates

**Figure 11:**
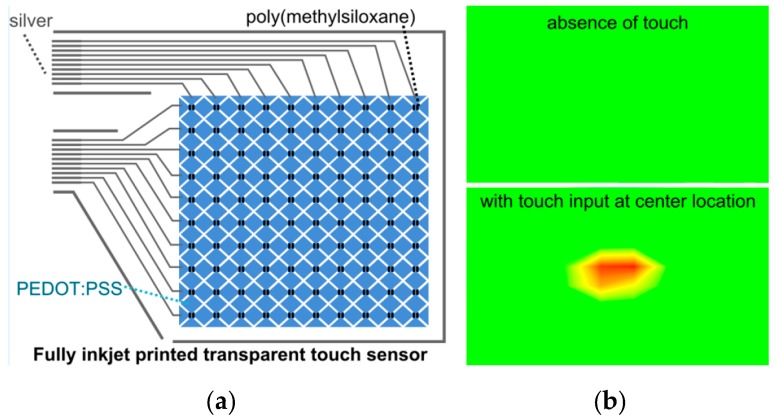
(**a**) Schematic of a fully inkjet-printed transparent touch sensor, a grid of PEDOT:PSS (x-axis and y-axis) and a poly(methylsiloxane) dielectric separator fabricated via inkjet printing; (**b**) Characterization of a touch sensor with the absence of touch and a single touch at the center (redrawn from [42]).

A fully inkjet-printed capacitive touch sensor is used to measure different applied pressures by characterizing the changes in capacitance [42]. PEDOT:PSS (Clevios P Jet V2, Heraeus Holding GmbH) is inkjet-printed on the substrate (borosilicate glass and PET) to obtain a grid of electrodes (in the horizontal and vertical directions). The diamond-shaped electrodes were connected with consecutive electrodes. To form insulating separators, a methylsiloxane dispersion filled with silica particles (Perma 6000, CA Hardcoating, Chula Vista, CA, USA), serving as dielectric ink was printed on each adjoining point of all the diamond-shaped electrodes. Ag nanoparticle ink (DGP 40LT- 15C, ANP) was used to form connections of the touch sensor, as shown in Figure 11a. A transparent polyethylene film (7524T11, McMaster) was laminated for structural protection and the performance of the touch sensor was evaluated using an integrated circuit board system (mXT1664S, Atmel, San Jose, CA, USA). Transparency of 85% was achieved by printing a single pass. The researchers also discovered that each pass contributed a 100-nm increase in the thickness of the deposited PEDOT:PSS layer, which tended to decrease in resistance with each increasing pass. The sensor accurately identifies single-touch and multi-touch events and a single touch event is shown in Figure 11b. 

We presented the salient features of inkjet-printed tactile sensors using various substrates. To give a brief overview about the resistance/resistivity of the inkjet-printed tracks using specific ink and substrate, we have been presented the Table 6. In our view point, resistance of the printed tracks can be an indicator of the “quality of the inkjet-printing.” Higher resistance could be due to imperfections such as, surface roughness, non-uniform ink deposition and/or degradation after sintering process.

## 4. Conclusions

We presented numerous research articles to demonstrate the fabrication processes and key features of inkjet-printed tactile sensors. Inkjet-printed electronics have attracted increasing research attention, as printed electronics have facilitated cost-effective fabrication. Humans like to interact with devices and gadgets in a convenient way. Touch sensing has arisen as a way to manipulate devices to enrich human-interaction. The integration of various technologies with different sensing mechanisms, materials and properties presents a considerable challenge. Artificial skin for robotic arms and bio-tactile sensors is still in an immature phase, although progress continues. In addition, the inexpensive and instant development of a highly sensitive, robust and reliable touch sensor remains a challenge. Inkjet-printing technology has been very helpful for solving these challenges by making the realization of touch sensors easier using unconventional substrates, advanced inks and newly devise sintering techniques. Therefore, advanced touch screens and sophisticated interactive-touch devices are now realizable and have become an essential part of daily life. 

## Figures and Tables

**Table 1:** Number of patents filed, organized by decade.

Year	US Patents	European Patents	Chinese Patents	International
1988–1997	57	1	0	1
1998–2007	147	1	2	1
2008–2017	276	4	47	177

Note: The term “touch sensor,” was used to query the Google patents search engine to collect these data. All kinds of patent types such as, design, utility, plant, defensive publications and additional improvements etc., are included.

**Table 2:** Comparison of glass, silicon, PDMS and paper as a sensor-substrate (redrawn from [21]).

Property	Glass	Silicon	PDMS	Paper
Surface profile	Very low	Very low	Very low	Moderate
Flexibility	No	No	Yes	Yes
Structure	Solid	Solid	Solid, gas-permeable	Fibrous
Sensitivity to moisture	No	No	No	Yes
Disposability	No	No	No	Yes
High-throughput fabrication	Yes	Yes	No	Yes
Functionalization	Difficult	Moderate	Difficult	Easy
Homogeneity of material	Yes	Yes	Yes	No
Price	Moderate	High	Moderate	Low
Initial investment	Moderate	High	Moderate	Low

**Table 3:** Dielectric properties of commonly used substrates.

Substrates	ε	tanδ	Ref. #
Kodak Photo Paper	2.85	0.05	[29]
PDMS	2.8	0.05	[30]
Kapton film	3	0.04	[31]
Cotton	1.6	0.04	[32]
Polyester	1.9	0.0045	[32]

**Table 4:** Conductivity and permittivity of most used metallic nanoparticle inks and dielectric inks, respectively.

Ink Material	Conductivity	Permittivity	Ref. #
Ag nanoparticle	0.4 to 2.5 × 10^7^ S/m	N/A	[29]
PVP dielectric ink	N/A	11.7	[25]
PEDOT:PSS	3 × 10^4^ S/m	N/A	[24]
Graphene	1 × 10^8^ S/m	N/A	[24]
SU-8 (dielectric layer)	N/A	4.2	[44]
Cu	5.96 × 10^7^ S/m	N/A	[45]
Au	4.42 × 10^7^ S/m	N/A	[45]

Note: N/A and N/S represent not applicable and not specified, respectively.

**Table 5:** Best resolution achieved using various inkjet-printing machines.

Ref. #	Nanoparticle Ink	Inkjet Printer	Substrate	Resolution	Sintering	Resistivity
[49]	In_2_O_3_	Electrohydrodynamic inkjet	Flexible plastic	2 µm	250 °C @ 150 min	N/S
[50]	Ag	Dimatix 2831	Si	2 µm	180 °C @ 30 min	N/S
[51]	Ag	Epson C110	Chrome paper	5 µm	160 °C @ 30 min	9.18 × 10^−8^–8.76 × 10^−8^ Ω·m

Note: The references showed in this table are not described in this review article, because their target applications are other than the tactile sensors. N/S represents not specified.

**Table 6:** Summary of ink materials, substrates and resistance/resistivity of the inkjet-printed capacitive tactile sensors presented in this review article.

Ref. #	Ink Material	Substrate	Resistance or Resistivity
[64]	Cu	Flexible plastic	200 mΩ/sq
[65]	Ag	Arylite	Resistance of AgNW film = 20 Ω
[67]	Ag	PDMS	Sheet Resistance of printed electrode = 19.2 ± 2.2 Ω/sq
[70]	Ag	Paper	Sheet Resistance = 0.19 Ω/sq
[71]	Ag	Paper	Sheet Resistance = 0.21 Ω/sq
[42]	PEDOT:PSS	Glass	Resistance across the ends of electrode = 6.9 K Ω

Note: Several inkjet-printed capacitive tactile sensors developed on various substrates have been discussed in Section 3 of this review article, however, only those articles for this table have been selected, which provide the value of resistance/resistivity.
